# Third Dentition

**Published:** 1849-01

**Authors:** 


In connection with the anomalous cases presented by Dr. Whitney, we take from the 1st No. Vol. VIII. American Journal and Library of Dental Science, a case headed Third Dentition, by the Baltimore Editor, which also presents nature in one of her capricious freaks, and is probably accounted for as well as the mysterious nature of these cases will admit.—[Cin. Ed.

### THIRD DENTITION.

About four years since, a lady aged about twenty-five years, wife of a medical gentleman of Baltimore, applied to us for an entire upper set of artificial teeth. The roots of the natural organs were still in the jaw. These we extracted, and at the expiration of nine months, the alveolar border having by this time undergone the changes which usually follow the loss of the teeth, we constructed and applied a dental substitute, which she still wears; but about two months since she again called on us, and playfully remarked, that she believed her jaw was about to be supplied with new teeth. On removing the artificial piece, we discovered, to our surprise, the crown of a right cuspidatus just peeping through the gum. Upon examination, it was found to be well developed, and very firmly attached to the jaw bone, but it has neither root nor socket.

Such anomalous productions have occasionally been met with before, but no attempt, that we are aware of, has ever been made to explain the manner of their formation. Do they originate from a tissue different from that which gives rise to the rudiments of the teeth of first and second dentition? Although this is a question which physiology has never attempted to solve, we think no one will affirm they do, inexplicable, apparently, as may otherwise seem the manner of their formation.

The rudiments of the teeth of first and second dentition, as is now universally admitted, are the product of mucous membrane, while those of third dentition, or the anomalous productions of which we are now speaking, would *seem* to originate from the periosteal tissue, if not from the bone itself.

In obedience to what law of developmental anatomy are they formed? Certainly to none primitively impressed upon the animal economy; for they have never been known to appear so long as the secondary teeth remain in the jaws. If the establishment of a law which governs the development of a part, depends upon a certain condition of other contiguous parts, it is possible, we may be able to explain the phenomena. But the admission of this assumption, would overthrow the theory which some have so ably and ingeniously maintained, that the rudiments of all the organs of the body exist at the very earliest period of intra-uterine existence, though perhaps not more fully than do the anomalous formations in question. It is very evident that certain parts, in certain states or conditions, and in particular locations, perform functions peculiar to the latter.

In other words, the condition and location of a part, determines the function or functions which it performs.

For example, when the mucous membrane along the course of the alveolar border, begins to assume a duplicated or grooved appearance, which it does at about the sixth week of intra-uterine existence, dental papillæ shoot up from it, and when, by similar duplication of this same tissue, behind the sacs of the temporary teeth, forming what Mr. Goodsir styles "cavities of reserve," the papillæ of the permanent teeth, one from the bottom or distal extremity of each duplication, begin to be developed. Hence, it would seem that this particular state or condition of this tissue, and in these particular locations was necessary to determine the development of teeth germs. This arrangement or condition of mucous membrane, in these particular locations, which always results from the development of the fœtus, may be sometimes produced by accidental causes, after all the organs of the body have attained their full size, or at any time during life; and, when it does occur, it is not unreasonable to suppose that a new tooth papillæ should be formed. Proceeding still farther, the development of a dental papillæ is the signal for the production of a dental follicle, which ultimately becomes a sac, and then an organ to supply the tooth, now considerably advanced in the process of formation, with a covering of enamel. But as the maxillary bone has previously attained its full size, it rarely, if ever, happens, that an alveolus is formed for these accidental productions, and consequently they seldom have roots, or if they do, they are very short and blunt. They are usually connected to the periosteum of the alveolar border, and this union is sometimes so close and intimate, that a very considerable force is necessary for their removal, or, at least, so far as our own observations go upon the subject, and we have had occasion to extract several in the course of our practice. As a general rule, however, they loosen in the course of a few years and drop out.

But it may be asked, how are such accidental duplications of the mucous membrane formed? This is a question, we admit, which it may not be easy to answer, satisfactorily, but we do not think it at all improbable, that they occur during the curative process that follows the removal of one or more teeth. The granulated walls of the gums surrounding an alveolus from which a tooth has been extracted, may become covered with this tissue before the socket is filled with a deposit of new bone, so as to prevent a complete union after they have come together, or, at any rate, of the surfaces of the duplicated membrane near the bone, and whenever such arrangement or condition of this tissue does take place, upon the alveolar border, and that it may occasionally, we think there can be no question, it is probable that a new tooth papillæ is produced, which, in the progress of its development, induces the formation of the various appendages necessary to the production of a perfect tooth.

This, in our opinion, is the only way that these fortuitous productions can be accounted for in accordance with true physiological principles. It is impossible to explain the manner of their formation in any other way. All must admit that the presence of mucous membrane is necessary to originate them, and we cannot conceive of any other way by which its presence beneath the general surface of the gums can be accounted for; but if we admit this explanation to be correct, the question is at once solved. We believe it is also owing to the accidental occurence of a certain arrangement or condition of the mucous membrane concerned in the production of the permanent teeth, consisting most likely of the formation of one or more "cavities of reserve," than is called for by the teeth of this dentition, that the development of supernumerary teeth are attributable.

In maintaining that the development of a tooth germ results from a certain state or condition of the mucous membrane investing the crest of the alveolar border, we are aware that we may be met with the inquiry, what determines this particular arrangement of this tissue, and the number of the teeth of first and second dentition? We reply, that, although we believe this question susceptible of satisfactory solution, we do not think it has any thing to do with the accidental production of these organs, if the fact be admitted that the papillary rudiments of the teeth originate from mucous tissue.

The operations of nature, it is true, are so secretly carried on, that we cannot see the precise *modus operandi* by which they are effected, yet, in the development of the various organs and structures of the body, we may see them in the various stages of their growth, as well as what precedes their arrival to these various stages in the progress of their formation, and upon which their accretion would seem to be dependent. The periods for the arrival of these stages of development, though somewhat irregular, occur for the most part in normal conditions of the body, at certain fixed epochs. Thus the papilla of the first temporary molar may usually be seen between the sixth and seventh weeks of intra-uterine existence, but previously to this time a slight groove or depression is observable in the mucous membrane of the part from whence it has its origin. The same is true with regard to the papillæ of all the other teeth, though the time for the commencement of their formation occurs at later periods. The peculiar change which takes place in the arrangement of the mucous tissue here, as well as the periods at which they occur, is doubtless determined by certain stages in the development of other parts, and these very likely may determine the established number which the teeth of both dentitions have.

If the foregoing views which we have advanced be correct, these fortuitous productions are not the result of a mere freak of nature, as they are sometimes facetiously styled. They are the result of the operation of an established law of the economy; and although, after the completion of the teeth of second dentition, its course is suspended, the occurrence of a similar arrangement or condition of the mucous tissue in the parts in question, will again put it in operation.

But time will not permit us, at present, to speculate at greater length upon this most curious and interesting subject. Should we have leisure to do so, we may resume the discussion of it at some future period, when we shall endeavor to present our thoughts upon the subject in a more clear and systematic manner. In the meantime, we should be glad to have an opportunity of laying before our readers the views of others upon the subject.

